# Post-stroke sensory hypersensitivity: insights from lesion-symptom and disconnection mapping

**DOI:** 10.1093/braincomms/fcaf176

**Published:** 2025-05-06

**Authors:** Hella Thielen, Nora Tuts, Lies Welkenhuyzen, Robin Lemmens, Alain Wibail, Irene M C Huenges Wajer, Christophe Lafosse, Dante Mantini, Céline R Gillebert

**Affiliations:** Department Brain and Cognition, Leuven Brain Institute, KU Leuven, Leuven 3000, Belgium; Department Brain and Cognition, Leuven Brain Institute, KU Leuven, Leuven 3000, Belgium; Department Brain and Cognition, Leuven Brain Institute, KU Leuven, Leuven 3000, Belgium; Department Psychology, Hospital East-Limbourgh, Genk 3600, Belgium; TRACE, Centre for Translational Psychological Research (TRACE), KU Leuven—Hospital East-Limbourgh, Genk 3600, Belgium; Department of Neurosciences, Experimental Neurology, KU Leuven, Leuven 3000, Belgium; Department of Neurology, University Hospitals Leuven, Leuven 3000, Belgium; Neurology, Hospital East-Limbourgh, Genk 3600, Belgium; Department of Medical Psychology, Amsterdam University Medical Centre, Amsterdam 1105AZ, The Netherlands; Paramedical and Scientific Director, RevArte Rehabilitation Hospital, Edegem 2650, Belgium; Department of Movement Sciences, Movement Control and Neuroplasticity Research Group, KU Leuven, Leuven 3000, Belgium; Department Brain and Cognition, Leuven Brain Institute, KU Leuven, Leuven 3000, Belgium; TRACE, Centre for Translational Psychological Research (TRACE), KU Leuven—Hospital East-Limbourgh, Genk 3600, Belgium

**Keywords:** sensory sensitivity, sensory overload, sensory processing

## Abstract

A post-injury increase in sensory sensitivity is frequently reported by acquired brain injury patients, including stroke patients. These symptoms are related to poor functional outcomes, but their underlying neural mechanisms remain unclear. Since stroke results in focal lesions that can easily be visualized on imaging, the lesions of stroke survivors can be used to study the neuroanatomy of post-injury sensory hypersensitivity. We used multivariate support vector regression lesion-symptom mapping and indirect structural disconnection mapping to uncover the lesion location and white matter tracts related to post-stroke sensory hypersensitivity. A total of 103 patients were included in the study, of which 47% reported post-stroke sensory hypersensitivity across different sensory modalities. The lesion-symptom and structural connectivity mapping identified the putamen, thalamus, amygdala and insula in the grey matter as well as fronto-insular tracts, and the fronto-striatal tract in the white matter as neural structures potentially involved in post-stroke sensory hypersensitivity. By examining the neuroanatomy of post-stroke sensory hypersensitivity in a large stroke sample, this study offers a significant advancement in our understanding of the neural basis of post-stroke sensory hypersensitivity.

## Teaser

Although post-stroke sensory hypersensitivity is a prevalent stroke symptom, its neural mechanisms remain unclear. Using multivariate lesion-symptom and disconnection mapping, this study identified the putamen, thalamus, insula, amygdala, fronto-insular tracts and the fronto-striatal tract as potentially involved in post-stroke sensory hypersensitivity.

## Graphical Abstract

**Graphical Abstract:**
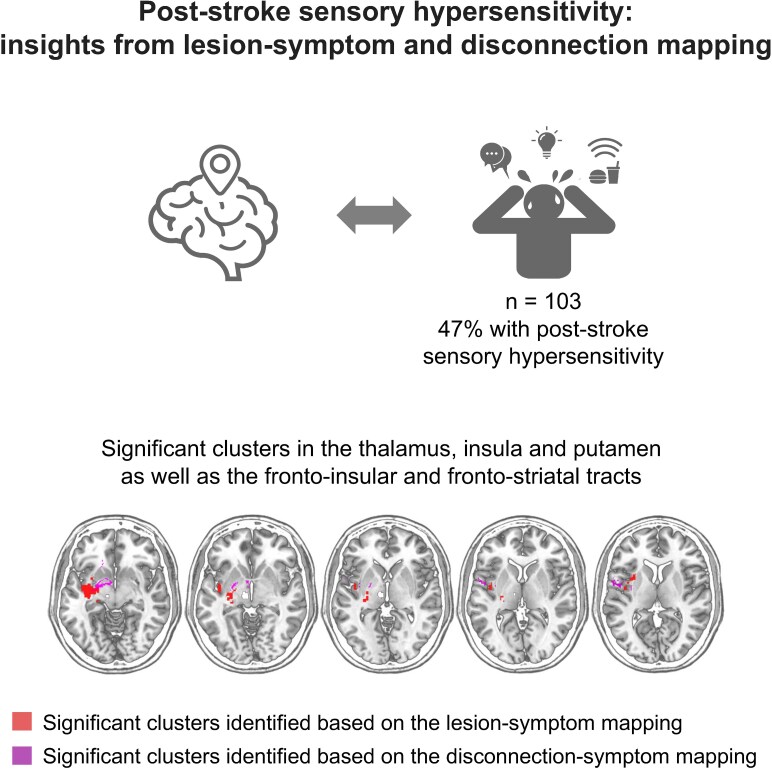

## Introduction

Successful participation in society depends on adequate processing of sensory rich environments (e.g. buying groceries in a busy supermarket, working in an open office, having a conversation at a family gathering). Individuals differ considerably in how sensitive they are to these sensory environments, ranging from a low to a high sensory sensitivity.^1,2^ Brain injuries, such as stroke, can affect an individual’s sensory sensitivity, thereby changing a person’s position along the sensory sensitivity continuum.^3,4^ Stroke, a disruption of blood supply to the brain that results in neural damage, can lead to increased sensory sensitivity, also termed post-stroke sensory hypersensitivity.^4,5^ Patients with post-stroke sensory hypersensitivity are easily overwhelmed by sensory rich environments, which can negatively impact their mental well-being, social functioning, and physical health.^6,7^ In a previous study, 76% of 204 chronic stroke patients reported post-stroke sensory hypersensitivity in either one (uni-modal post-stroke sensory hypersensitivity) or multiple sensory modalities (multi-modal post-stroke sensory hypersensitivity).^4^ Notably, post-stroke sensory hypersensitivity can also occur in the subacute phase after stroke.^6^

These symptoms are not specific to stroke patients but are also seen after other types of acquired brain injury (traumatic brain injury, brain tumours), in the neurotypical population, and in several clinical populations, including individuals with autism spectrum disorder, attention-deficit/hyperactivity disorder (ADHD), or chronic pain.^4,8-11^ Across these populations, the underlying mechanisms contributing to self-reported sensory sensitivity remain largely unknown. More specifically, it is uncertain whether inter-individual differences in subjective (self-reported) sensory sensitivity are related to inter-individual differences in behavioural (i.e. the ability to detect or discriminate between different sensory stimuli) or neural sensory sensitivity (i.e. the neural response to sensory stimuli).^2^ Characterizing the underlying behavioural and neural mechanisms of subjective sensory sensitivity is necessary for developing rehabilitation protocols that can limit the negative impact of high sensory sensitivity on daily functioning.

Stroke patients are ideal candidates for studying the neural basis of sensory hypersensitivity after acquired brain injury since stroke typically results in focal lesions that can be easily visualized on routine clinical imaging. In a previous systematic review that investigated the neuroanatomy of post-stroke sensory hypersensitivity,^6^ we described four case studies that linked uni-modal post-stroke sensory hypersensitivity (hypersensitivity to visual, auditory, gustatory, olfactory stimuli) to insular damage.^12-15^ We complemented these results with a multiple case study describing three right-hemispheric stroke cases with multi-modal post-stroke sensory hypersensitivity whose lesions overlapped in the right anterior insula, the claustrum, and the Rolandic operculum.^6^

However, the results of the systematic review and the multiple case study might be biased. On the one hand, the sample of our multiple case study was limited to patients with self-reported post-stroke sensory hypersensitivity after right-hemispheric damage. On the other hand, both the systematic review and multiple case study only included patients with self-reported post-stroke hypersensitivity without comparing their lesion locations to those of patients without post-stroke sensory hypersensitivity. This comparison is important, as the insula is frequently affected following a middle cerebral artery stroke.^16^ To mitigate these limitations, the brain lesions of left- and right-hemispheric patients with and without post-stroke sensory hypersensitivity should be compared to investigate which region, when damaged, could result in post-stroke sensory hypersensitivity. Lesion-symptom mapping is a powerful technique that examines the relationship between behaviour and brain damage without a priori defining a region of interest or excluding patients with or without certain behavioural profiles.^17^ Traditionally, univariate lesion symptom mapping techniques, compute, per voxel, if there is a statistically significant difference in behaviour between patients with and without damage in that voxel.^18^ However, research has shown that multivariate lesion-symptom mapping has a higher sensitivity and specificity for identifying lesion-behaviour relationships than univariate (voxelwise) approaches.^19^ In contrast to univariate approaches, multivariate lesion-symptom mapping considers the effect of all lesioned voxels simultaneously and acknowledges intervoxel correlations (i.e. some voxels are often damaged together due to the non-random distribution of lesions across the brain) when modeling lesion-behaviour relationships. Although multivariate lesion-symptom mapping offers a topological approach that identifies specific grey matter regions that are necessary for certain functions, it does not consider that brain lesions can have structural and functional impacts on non-damaged parts of brain networks.^20^ In addition, since white matter tracts are spatially distributed, a disconnection at different locations among this tract can have similar behavioural consequences.^21^ White matter integrity can be directly assessed using Diffusion Tensor Imaging but this technique is hard to implement in a large patient sample due to its reliance on high-quality nonclinical brain imaging.^22,23^ To overcome these limitations, indirect structural disconnection mapping can be used to map individual lesions (normalized to a common template) onto a database of structural networks in neurologically healthy adults to estimate the disruptions in white matter integrity caused by the lesion.^22,24,25^

This study aimed to investigate the neuroanatomy of post-stroke sensory hypersensitivity in a first-ever subacute stroke sample using multivariate lesion-symptom and disconnection mapping. Learning more about the neuroanatomy of post-stroke sensory hypersensitivity can greatly enhance our understanding of the underlying mechanisms of these subjective symptoms as well as help identify patients that are at risk of developing post-stroke sensory hypersensitivity.

## Materials and methods

### Participants

Stroke patients were recruited between December 2019 and January 2023 from the acute stroke unit of University Hospitals Leuven and the rehabilitation units of RevArte Rehabilitation Hospital and Hospital East-Limburg. Recruitment was halted between March 2020 and June 2020 due to the COVID-19 pandemic. Stroke patients were included when (1) they were able to provide informed consent, (2) they were adult (aged 18 years or older), (3) they completed a patient-friendly sensory sensitivity questionnaire [the Multi-modal Evaluation of Sensory Sensitivity (MESSY)], (4) they were first-ever stroke survivors, (5) at least one clinical brain scan was available, and (6) the stroke lesion was visible on clinical imaging. Additional exclusion criteria were (1) the presence of major microvascular damage (defined as Fazekas grade 3),^26^ (2) having a subdural or subarachnoid haemorrhage, (3) presence of Wallerian degeneration, (4) having a pre-existing neurological disorder (previous traumatic brain injury, stroke, tumour), (5) having a formal diagnosis of autism spectrum disorder, ADHD or schizophrenia, and (6) having another psychiatric disorder (e.g. post-traumatic stress disorder) that could impact their sensory sensitivity. We did not exclude patients based on their lesion location, cognitive profile or time since stroke.

## Materials

### Multi-modal evaluation of sensory sensitivity

The MESSY is a patient-friendly questionnaire that assesses the sensitivity to sensory stimuli across several modalities (i.e. multisensory, visual, auditory, tactile, olfactory, gustatory and motion sensitivity as well as sensitivity to environmental temperature).^4^ Multisensory sensitivity refers to the sensitivity to stimuli from different sensory modalities that are present at the same time (e.g. the simultaneous presence of visual, auditory, olfactory and gustatory stimuli in a restaurant). Per modality, the MESSY assesses whether patients experience an increase in their sensory sensitivity after their brain injury using open questions (i.e. ‘Since your brain injury, have you become more sensitive to sounds? How did you notice this or in which situations did you notice this?’). These open questions are used to determine whether high sensory sensitivity was linked to stroke onset (i.e. to differentiate post-stroke symptoms from pre-existing sensory hypersensitivity). In addition, the MESSY uses 30 multiple-choice items which are answered on a five-point Likert-scale (ranging from never/not at all to very often/extremely). The multiple-choice items are summed to assess the severity of the sensory sensitivity per modality or across all modalities (i.e. total score of the MESSY). The MESSY is an aphasia-friendly questionnaire, since it uses pictograms, places one item per page, and displays key concepts in a question in bold.^27^ In this study we used the pen-and-paper version of the MESSY that was developed for an inpatient acquired brain injury population.

The open-ended questions of the MESSY are used to assess whether stroke patients experience post-stroke hypersensitivity, this is an increase in their sensory sensitivity since their stroke. If stroke patients indicated that they experienced a post-stroke increase in their sensitivity to one or multiple sensory modalities on the open-ended questions of the MESSY, they were considered as having post-stroke sensory hypersensitivity. Accordingly, patients in the group without post-stroke sensory hypersensitivity reported no post-stroke increase in their sensory sensitivity to any sensory modality. The multiple-choice items of the MESSY assess the current level of sensory sensitivity. Since patients with post-stroke sensory hypersensitivity can differ in their pre-morbid levels of sensory sensitivity, these total scores cannot be used to discern the severity of post-stroke changes in sensory sensitivity without having a pre-morbid reference point.

### The Oxford cognitive screen—NL

To assess post-stroke cognition, we administered the Dutch version of the Oxford Cognitive Screen (OCS) (version A).^28^ This cognitive screening tool consists of 11 subtests assessing visual field deficits and various cognitive domains such as attention, memory, language, praxis, and numeracy. In contrast to other commonly used screening tools (such as the Montreal Cognitive Assessment), the OCS provides domain-specific test scores and is thought to be aphasia- and neglect-friendly.^29^ The parallel-form reliability and convergent validity of the OCS were deemed satisfactory by previous studies.^29,30^

### Structural anamnesis

During a structural anamnesis participants answered questions regarding several demographic variables (i.e. their age, sex, education level) and their medical background. Stroke type, time since stroke, the number of previous strokes, and the score on the National Institute of Health Stroke Scale (NIHSS)^31^ were gathered from the electronic medical files of the stroke patients. The NIHSS score signifies the severity of stroke-related neurological deficits with higher scores indicating more severe deficits. The NIHSS is used to assess level of consciousness, visual field deficits, as well as somatosensory, motor and language deficits.

### Procedure

This study is part of a larger study assessing post-stroke sensory sensitivity. Ethical approval for this study was granted by the Medical Ethics Committee of the Hospital of East-Limburg (application number: CTU2019055), the Ethics Committee Research UZ/KU Leuven (application number: S63063), and Medical Ethics Committee of the GasthuisZusters Hospital Antwerp (application numbers: 190904ACADEM). Participation consisted of three sessions, which were completed in a distraction-free room. During the first session written informed consent was obtained in accordance with the World Medical Association Declaration of Helsinki. Afterwards, participants completed the MESSY and the structural anamnesis. Clinical imaging was acquired from the electronic medical files of the stroke patients. During the three sessions, that lasted ∼60 min each, patients completed additional neuropsychological tasks and questionnaires that are beyond the scope of the current study. In patients who completed all three sessions, there were on average 9 days between the first and the third session (range: 2–28 days).

### Data analysis

Analyses were conducted in R (version 4.2.2)^32^ and Matlab2024b.^33^ Figures were created using Adobe Photoshop (2020).

#### Behavioural data analysis

During the analyses of behavioural data, alpha was set to 0.05 and all reported *P* values were corrected for multiple comparisons using the Holm method.^34^

#### Lesion delineation and pre-processing

Lesions were delineated manually on the axial plane of a clinical brain scan (Fluid Attenuated Inversion Recovery (FLAIR): *n* = 46, Diffusion Weighted Imaging (DWI): *n* = 37, Computed Tomography (CT): *n* = 20) using MRIcron and a Wacom Cintiq Pro tablet by trained investigators (HT, NT) (for details see Table 1). A recent study indicated that there was no evidence for a difference in accuracy between CT- and MRI-based lesion delineation.^35^ If multiple brain scans were available for one patient, the scan used for lesion delineation was selected following the procedure outlined by Biesbroek *et al*.^36^ We used SPM12 (https://www.fil.ion.ucl.ac.uk/spm) to smooth the lesion masks at 8 mm full width half maximum, resliced them to 2 mm isotropic voxels, and normalized them to Montreal Neurological Institute (MNI) space by applying a non-linear deformation calculated on the brain scan using the ‘old segment’ function. All normalized lesion masks were visually inspected by comparing them both with the normalized brain scan and with a template image in MNI space. If a small lesion focus was removed due to smoothing, this focus was manually added to the normalized lesion map.^36-38^ Descriptive lesion overlap maps were created in MRIcron by superimposing individual lesion maps onto axial slices of the T1-weighted template from the MNI (ch2-template).

**Table 1:** The resolution of the included scans per scan type

Scan type	*n*	Mean voxel size [Range] (in mm)		
		X	Y	Z
FLAIR	46	1.52 [0.49–6.45]	0.84 [0.45–4.01]	1.34 [0.46–6.5]
DWI	37	0.99 [0.57–1.2]	2.28 [0.57–6.43]	3.28 [0.9–6.48]
CT	20	1.04 [0.33–2.99]	0.52 [0.32–2.61]	1.71 [0.32–3]

#### Multivariate lesion-symptom mapping

To compare the lesion location of patients with and without post-stroke sensory hypersensitivity, we performed a support vector regression-based multivariate lesion-symptom mapping (SVR-LSM)^19^ using the SVR-LSM toolbox.^39^ SVR-LSM uses machine learning and SVRs (with a radial basis function) to compute, for each voxel, a feature weight (a beta value) that represents the strength of the relationship between that voxel and the behaviour of interest. Since these feature weights cannot be interpreted directly, permutation testing is used to assess their statistical significance. In line with recommendations from Zhang *et al*.^19^ the hyperparameter values of the machine learning algorithms were set a priori at a cost of 30 and a gamma of 5. We did not perform hyperparameter optimization to avoid overfitting, which can lead to unstable, non-generalizable models.^40-42^ Only voxels that were lesioned in at least five participants (5% of the sample) were considered in the analysis. To control for multiple comparisons, we used a permutation-based continuous family wise error correction (with 2000 permutations, *P* = 0.05, and *v* = 10) which permitted 10 false positive voxels (similar to Faulkner and Wilshire^43^; Mirman *et al*.^44^). To control for the effects of lesion volume and age, we regressed lesion volume out of the behavioural scores and lesion maps (following the recommendations of DeMarco and Turkeltaub^39^) and included age as a covariate, after standardizing it. Anatomic labelling was performed using the Automated Anatomical Labelling Atlas 3.^45^

#### Indirect structural disconnection mapping

To compare white matter integrity between patients with and without post-stroke sensory hypersensitivity, we conducted a disconnection analysis using the Disconnection maps software of the BCBtoolkit (http://toolkit.bcblab.com).^24^ This software estimates the probability of disconnection for each voxel by projecting individual lesion maps onto existing white matter atlases generated from 7T DWI data in 179 neurotypical adults.^46^ The lesion maps of the stroke patients served as a seed region for tractography, allowing us to identify disrupted voxels. We classified a voxel as disconnected, when the probability of disconnection was above 50%. Descriptive disconnection overlap maps were created by superimposing individual disconnection maps onto axial slices of the T1-weighted template from the MNI (ch2-template). For the statistical analysis, we applied the same SVR approach used previously, by entering the disconnection maps as independent variables instead of the lesion maps (similar to Weaver *et al*.^47^). The analysis was restricted to voxels that were disconnected in at least five patients, permutation testing (with 2000 permutations) was performed to assess the significance of the results, (standardized) age was added as a covariate, and we controlled for total disconnection volume by regressing it out of both the behavioural score and the lesion data. Labelling of disconnected white matter tracts was done by using the white matter atlas of Rojkova *et al*.^46^ and the JHU white matter atlas implemented in MRIcron.

## Results

### Participants

**Figure 1:**
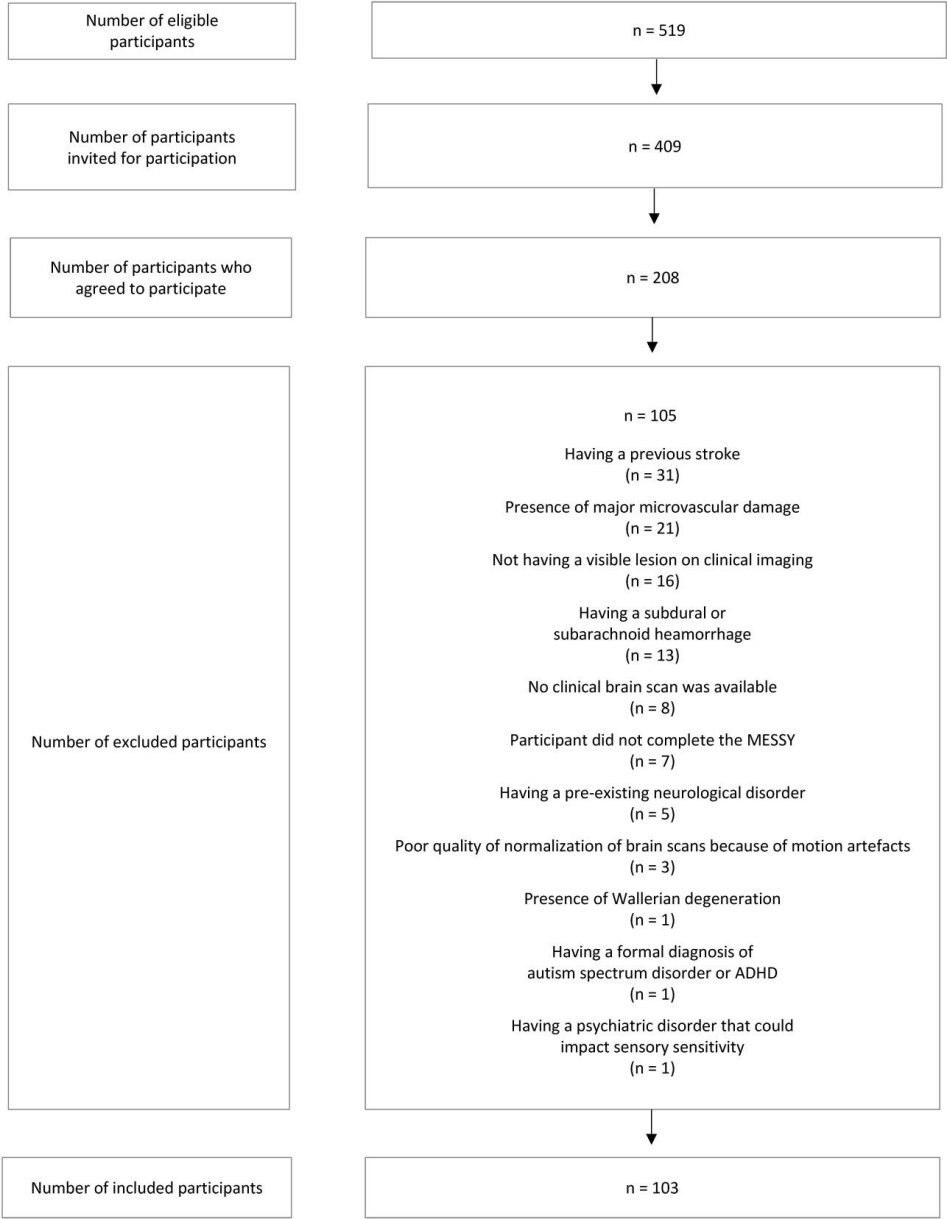
**Flowchart of participant recruitment and inclusion**. Of 519 eligible individuals, 409 were invited and 208 agreed to participate. After exclusions for reasons such as prior stroke, major microvascular damage, no visible lesion on imaging, haemorrhage, missing or poor-quality scans, and neurological or psychiatric conditions, 103 participants were included in the final analysis. ADHD, attention-deficit/hyperactivity disorder; MESSY, multi-modal evaluation of sensory sensitivity.

Of the 208 stroke patients that participated in the study, 103 patients were included in the analyses (see Fig. 1 for the participant flow chart). Scans were acquired on average 6 days after stroke (standard deviation: 12) and there were on average 17 days between acquisition of the scan and completion of the MESSY (standard deviation: 26). Of the 103 included patients, 55 patients (53%) were recruited in an acute stroke unit and 48 (47%) in a rehabilitation setting.

The majority of the included stroke patients (77%) had an ischemic stroke. Forty-eight stroke patients (48% of the final sample) reported a post-stroke increase in their sensitivity to sensory stimuli. The characteristics of stroke patients with and without post-stroke sensory hypersensitivity are displayed in Table 2 (for details see Supplementary Table 1). There was no evidence for a significant difference between patients with and without post-stroke sensory hypersensitivity in age, lesion volume, days between stroke onset and clinical imaging, days between stroke onset and MESSY completion, the proportion of patients who completed higher education (Fisher’s exact test: Holm adjusted *P* = 1), NIHSS score, and cognitive performance as assessed using the OCS-NL (see Supplementary Tables 1 and 2). Furthermore, in patients with post-stroke sensory hypersensitivity there was no evidence for a relationship between the sensory sensitivity severity (the total score of the MESSY) on the one hand, and lesion volume (Spearman rho: −0.18, Holm adjusted *P* = 1), age (Spearman rho: 0.01, Holm adjusted *P* = 1), sex (Wilcoxon test: W: 171, Holm adjusted *P* = 0.23), NIHSS score (Spearman rho: −0.24, Holm adjusted *P* = 1), days between stroke onset and clinical imaging (Spearman rho: −0.26, Holm adjusted *P* = 0.67) and days between stroke onset and MESSY completion (Spearman rho: −0.09, Holm adjusted *P* = 1) on the other hand.

**Table 2:** Characteristics of the patients with and without post-stroke sensory hypersensitivity (*n* = 103)

	Patients without post-stroke sensory hypersensitivity	Patients with post-stroke sensory hypersensitivity
Number of patients	55	48
Age: mean (SD), in years	69 (12)	62 (15)
Age range: in years	29–89	26–90
Number of male patients (%)	35 (64%)	28 (58%)
Number of patients who completed higher education^a^ (%)	15 (28%)	19 (40%)
Number of patients with an ischemic/haemorrhagic stroke (%)	48 (87%)/7 (13%)	35 (73%)/13 (27%)
Lesioned hemisphere: left/right/bilateral: number of patients (%)	23 (42%)/26 (47%)/6 (11%)	21 (44%)/25 (52%)/2 (4%)
Lesion volume: mean (SD), in cc	41 (53)	36 (53)
Time between MESSY completion and clinical imaging: mean (SD), in days	11 (12)	26 (33)
Time between stroke onset and clinical imaging: mean (SD), in days	4 (8)	7 (16)
MESSY Total Score Possible range [30–150]	44 (13)	65 (17)
NIHSS score: mean (SD)	4 (3)	5 (4)

SD, standard deviation. Higher education: at least a bachelor degree awarded by a college or university. Cc, cubic centimetre. *P* values were adjusted for multiple comparisons using a Holm correction.^34^ There were on average 9 days between MESSY completion and the completion of the NIHSS. The NIHSS scores of 17 patients with post-stroke sensory hypersensitivity and five patients without post-stroke sensory hypersensitivity were missing. For a detailed version of this table see Supplementary Table 1.

^a^The education level of one patient without post-stroke sensory hypersensitivity and one patient with post-stroke sensory hypersensitivity was not reported.

Thirty-seven of the 48 patients with post-stroke sensory hypersensitivity (77%) reported experiencing multi-modal post-stroke sensory hypersensitivity: their increase in sensory sensitivity was present in more than one sensory modality. The number of patients who experienced an increased sensitivity per sensory modality as well as a description that participants gave to describe their heightened sensitivity to that sensory modality are provided in Table 3. The total MESSY score of patients with post-stroke sensory hypersensitivity (i.e. the severity of sensory sensitivity) was significantly higher as compared to stroke patients without post-stroke sensory hypersensitivity (see Supplementary Table 1).

**Table 3:** The number of patients with post-stroke sensory hypersensitivity for a specific modality as well as examples of descriptions patients gave to describe their symptoms

Sensory modality	Number of patients with post-stroke sensory hypersensitivity for a specific modality	Examples of descriptions patients gave to describe their symptoms
Multisensory	33	*‘I get overwhelmed during my physical therapy. I feel like there is too much happening all at once (listening to my therapist, other people moving around me, the sunlight that shines through the windows, and the radio that is on).’* ‘*I detest having visitors: it makes me feel anxious and stressed when there are too many people around me. Before my stroke I was very social.’*
Visual	29	*‘Since my stroke I started disliking bright sunlight and fast moving images on the television.’*
Auditory	21	*‘I notice that I experience typical sounds, such as the sound of my playing grandchildren or music, as highly aversive. Being surrounded by these sounds gives me a headache and makes me feel exhausted.’*
Motion	16	*‘When I am seated in a moving car or when I am driven around in my wheelchair, it feels like everything around me is moving. This makes me incredibly nauseous and feels very unstable (like I am going to tip over).’*
Environmental temperature	11	*‘I get overwhelmed by the slightest increase in temperature.’*
Olfactory	8	*‘My sense of smell has increased since my stroke. Smells of detergent or makeup are much more intense than before more stroke.’*
Gustatory	2	*‘Sweet or sour foods taste incredibly intense. I have stopped eating certain foods due to this increase in taste.’*
Tactile	1	*‘Since my stroke I feel overwhelmed by brushing my hair (when the comb lightly touches my scalp) or when my wife touches my arm. I avoid physical contact.’*

Patients who reported multi-modal post-stroke sensory hypersensitivity are counted multiple times in this table. Sensory modalities were ordered from most to least prevalent.

### Multivariate lesion-symptom mapping

**Figure 2:**
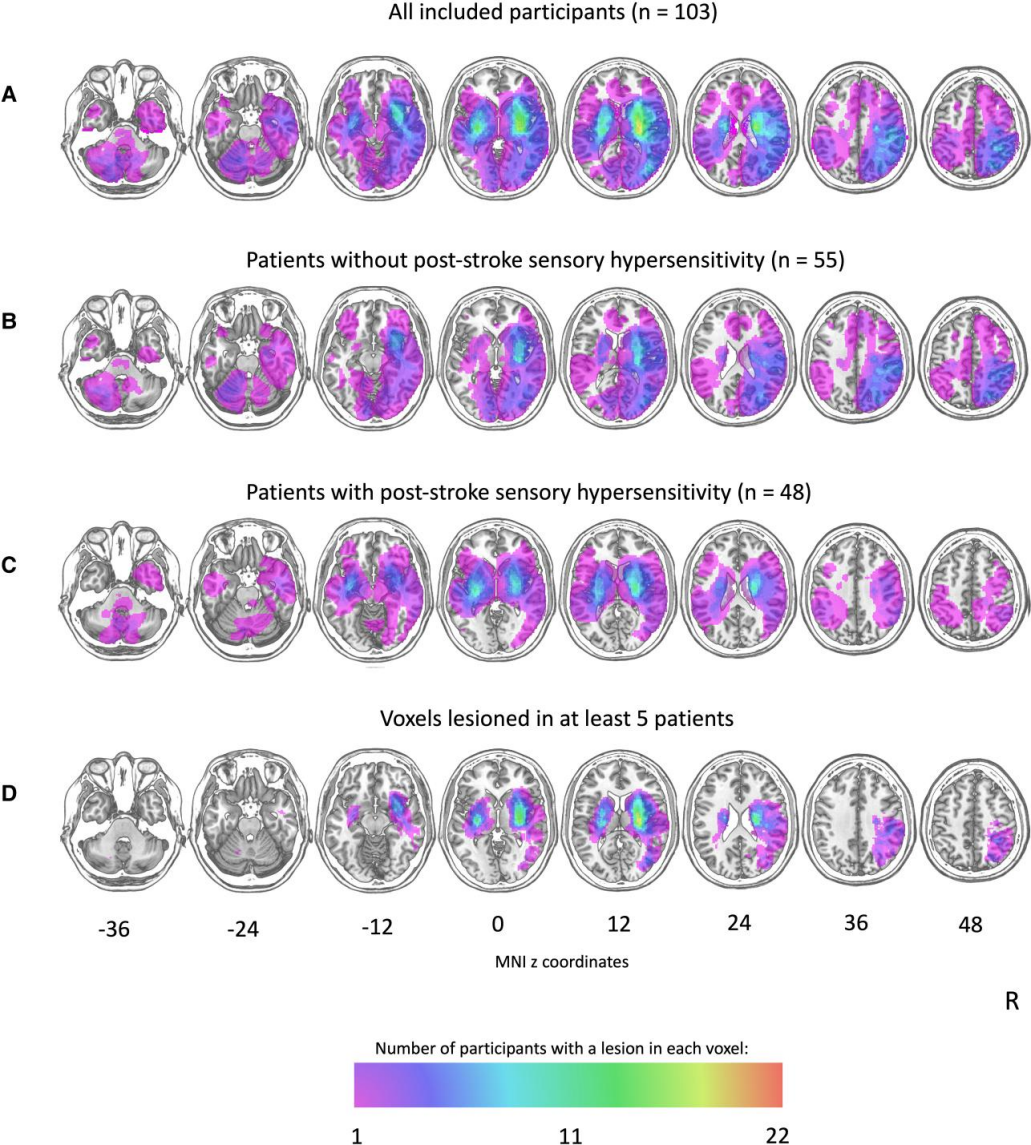
**Lesion overlap maps of patient with and without post-stroke sensory hypersensitivity**. (**A**) Lesion overlap map (descriptive) of all included participants (*n* = 103), created by superimposing individual lesion maps onto axial slices of the T1-weighted template from the MNI (ch2-template). (**B**) Lesion overlap map (descriptive) of patients without post-stroke sensory hypersensitivity (*n* = 54). (**C**) Lesion overlap map (descriptive) of patients with post-stroke sensory hypersensitivity (*n* = 49). (**D**) Lesion overlap map displaying the voxels that were lesioned in at least five patients, i.e. the voxels that were included in the statistical analysis. The lesion maps are visualized on axial slices of the T1-weighted template from the MNI (ch2-template). The numbers refer to the MNI coordinates of the *z*-axis. The color scale indicates the number of patients with a lesion in a specific voxel. The right hemisphere is presented on the right side.

**Figure 3:**
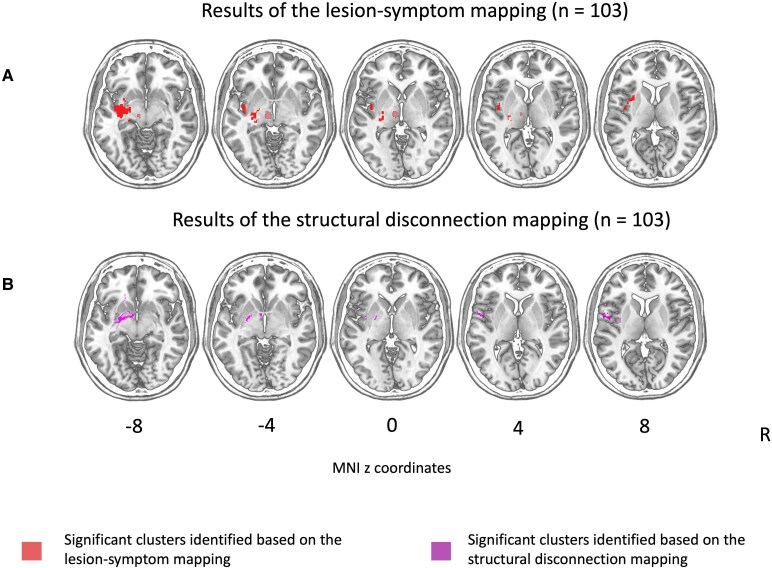
**Results of the lesion-symptom and structural disconnection mapping (*n* = 103)**. Statistically significant voxels identified by (**A**) the support vector regression-based multivariate lesion symptom mapping showing a relationship between post-stroke sensory hypersensitivity and the left insula, thalamus, putamen and amygdala based on the lesion maps; and (**B**) structural disconnection mapping showing a relationship between post-stroke sensory hypersensitivity and the fronto-insular tracts 3,4 and 5 as well as the fronto-striatal tract. In both analyses a continuous family wise error correction for multiple comparisons was applied and the results were corrected for the effects of age and lesion or disconnection volume. The lesion maps and white matter tracts are visualized on axial slices of the T1-weighted template from the MNI (ch2-template). The numbers refer to the MNI *z*-coordinates of the corresponding slices. The right hemisphere is presented on the right side. The SVR-LSM identified a relationship between post-stroke sensory hypersensitivity and clusters of voxels in the left insula, thalamus, putamen and amygdala based on the lesion maps.

Figure 2 shows an overlay of the lesions (for the entire sample, and the patients with and without post-stroke sensory hypersensitivity separately) as well of the voxels that were included in the analysis (i.e. voxels that were lesioned in at least five participants). The SVR-LSM identified a relationship between post-stroke sensory hypersensitivity and clusters of voxels in the left insula, thalamus, putamen and amygdala based on the lesion maps (see Fig. 3 and Table 4). To test whether these left-hemispheric results were merely due to a lack of left-hemispheric control data, we conducted a supplementary analysis where we flipped left-hemispheric lesions (or the left-hemispheric part of bilateral lesions) onto the right hemisphere (similar to Meyer *et al*.^48^ and Wang *et al*.^49^) (see Supplementary Analysis 1 and Supplementary Fig. 1). The SVR-LSM on the flipped lesions identified significant clusters in the thalamus and putamen (Supplementary Table 3 and Supplementary Fig. 2).

**Table 4:** Descriptive statistics of the significant clusters identified by SVR-LSM

	Number of voxels	MNI centre of mass coordinates			Peak *Z* value in MNI coordinates	Anatomical location
		X	Y	Z	Z	
Cluster 1	428	−32	−8	−3	−18	Left Putamen, Insula, Amygdala
Cluster 2	79	−9	−16	−3	−8	Left Thalamus

Anatomical location was determined using the Automated Anatomical Labelling Atlas 3.^45^

### Indirect structural disconnection mapping

**Figure 4:**
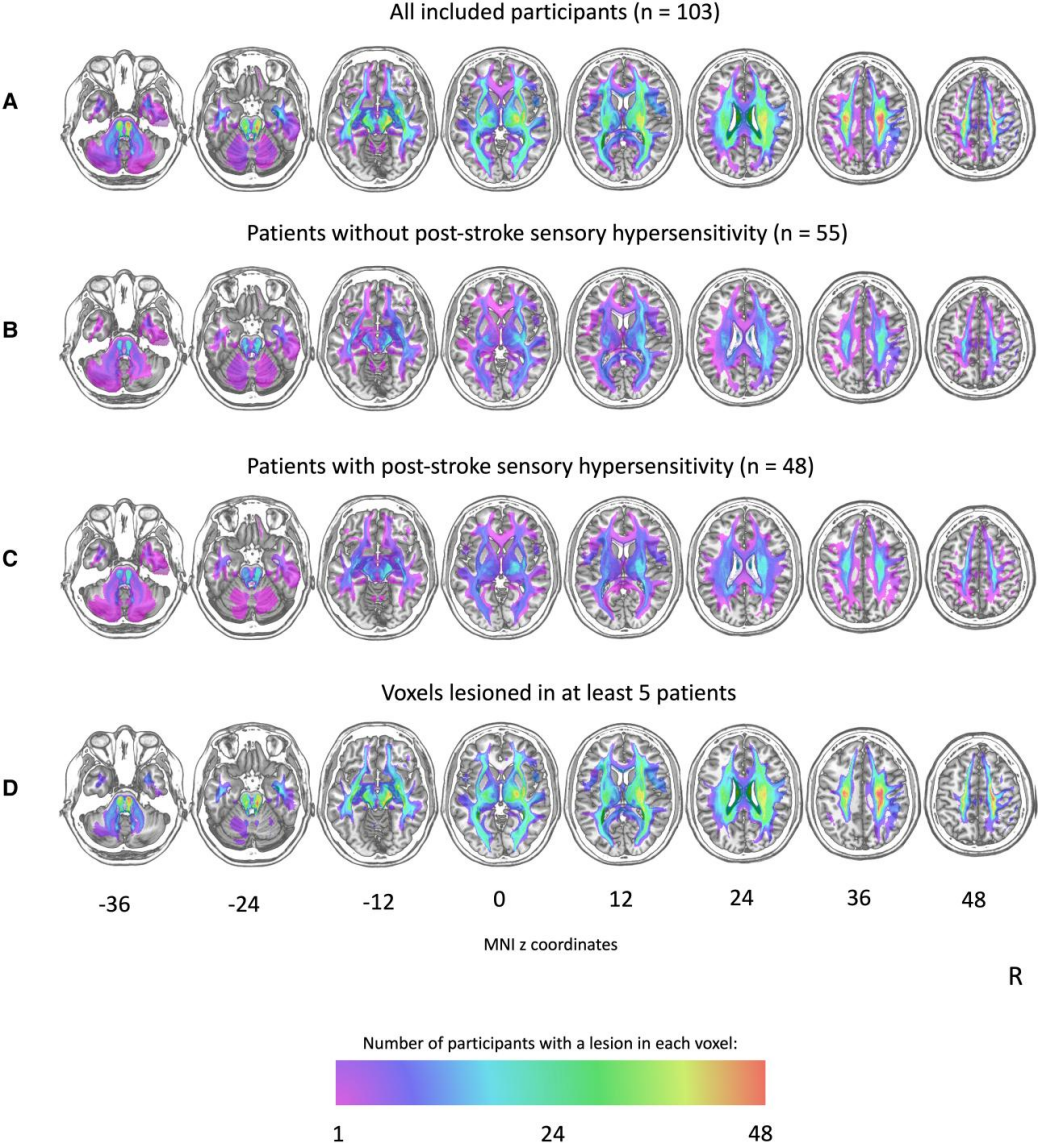
**Disconnection overlap maps of patient with and without post-stroke sensory hypersensitivity**. (**A**) Disconnection overlap map (descriptive) of all included participants (*n* = 103), created by superimposing individual disconnection maps onto axial slices of the T1-weighted template from the MNI (ch2-template). (**B**) Disconnection overlap map (descriptive) of patients without post-stroke sensory hypersensitivity (*n* = 54). (**C**) Disconnection overlap map (descriptive) of patients with post-stroke sensory hypersensitivity (*n* = 49). (**D**) Disconnection overlap map displaying the voxels that were lesioned in at least five patients, i.e. the voxels that were included in the statistical analysis. The lesion maps are visualized on axial slices of the T1-weighted template from the MNI (ch2-template). The numbers refer to the MNI coordinates of the *z*-axis. The color scale indicates the number of patients with a lesion in a specific voxel. The right hemisphere is presented on the right side.

Figure 4 shows an overlay of the disconnection maps (for the entire sample, and the patients with and without post-stroke sensory hypersensitivity separately) as well of the voxels that were included in the analysis (i.e. voxels that were lesioned in at least five participants). The structural disconnection mapping identified a relationship between post-stroke sensory hypersensitivity and the fronto-insular tracts 3,4 and 5 as well as the fronto-striatal tract (see Fig. 3 and Table 5).

**Table 5:** Descriptive statistics of the significant clusters identified by the structural disconnection mapping

	Number of voxels	MNI centre of mass coordinates			Peak *Z* value in MNI coordinates	White-matter tract
		X	Y	Z	Z	
Cluster 1	1181	−17	1	−9	−31	Frontal-striatal tract
Cluster 2	795	−47	−2	10	−1	Frontal-insular tracts 3,4 and 5

Anatomical location was determined using white matter atlas of Rojkova *et al*.^46^ and the JHU white matter atlas implemented in MRIcron.

## Discussion

The aim of this study was to examine the neuroanatomy of post-stroke sensory hypersensitivity (i.e. an increase in sensory sensitivity after stroke) in a subacute stroke sample using state-of-the-art techniques. We found evidence for a relationship between post-stroke sensory hypersensitivity and damage to the insula as well as disconnection of fronto-insular tracts (see Fig. 3, Tables 4 and 5). This corresponds with previous case studies that described uni- or multi-modal post-stroke sensory hypersensitivity after insular damage as well as fMRI research linking insula activation and sensory hypersensitivity in fibromyalgia patients.^6,9,12-14^ In addition, our results suggest an association between post-stroke sensory hypersensitivity and damage to other structures such as the thalamus, putamen, amygdala and frontal-striatal tracts (see Fig. 3, Tables 4 and 5), which corresponds to previous fMRI research linking the amygdala and thalamus to sensory hypersensitivity in children with ASD.^50,51^ This study provides a significant advancement in our understanding of the neural basis of post-stroke sensory hypersensitivity as it studied the neuroanatomy of these symptoms in a large stroke sample.

### The neural basis of post-stroke sensory hypersensitivity

As empirical evidence regarding the underlying behavioural mechanisms of post-stroke sensory hypersensitivity is limited,^3,50,52^ the association between post-stroke sensory hypersensitivity on the one hand and lesions to the insula, amygdala, thalamus, putamen, fronto-insular and frontal-striatal tracts on the other hand can be understood through prominent hypotheses regarding the underlying cognitive and psychological mechanisms of sensory hypersensitivity after acquired brain injury.

The first hypothesis, the information-processing hypothesis of sensory hypersensitivity, posits that impaired selective attention might underlie sensory hypersensitivity symptoms.^53,54^ This hypothesis is supported by empirical evidence for a relationship between sensory hypersensitivity and selective attention in neurotypical adults and stroke patient with post-stroke visual hypersensitivity.^55,56^ In this context, the involvement of insula, putamen, thalamus and frontal-striatal tract can be explained by their established roles in sensory filtering. The insula is a key hub in the salience network and plays a pivotal role in identifying and filtering relevant sensory stimuli.^57^ The thalamus, in turn, is seen as a relay station that receives incoming sensory information from different senses and selects information to send to the cortex for further processing.^58^ Higher cortical regions project onto the thalamus to drive this sensory filtering towards goal-directed information.^59,60^ One of these feedback loops projects from the prefrontal cortex to the thalamus through the basal ganglia, suggesting that damage to fronto-striatal tracts could disrupt adaptive attentional allocation.^61^

In contrast to this hypothesized relationship between selective attention and post-stroke sensory hypersensitivity, it must be noted that there was no difference in performance on attention-based pen and paper tests of the OCS between patients with and without post-stroke sensory hypersensitivity (see Supplementary Table 2). However, as the OCS is a cognitive screen as well as a pen-and-paper-based measure, its sensitivity to pick up subtle attentional impairments might be limited.^62-64^ Indeed, using a computerized assessment of visual attention, we have previously found that post-stroke visual hypersensitivity is related to selective attention impairments.^65^

In addition to a relationship between selective attention and post-stroke sensory hypersensitivity, researchers have also proposed an involvement of psychological mechanisms. Specifically, sensory hypersensitivity might be driven by anxiety-related hypervigilance.^53^ Heightened anxiety after stroke may be secondary to the traumatic experience of stroke onset, but can also be a primary result of damage to the thalamus, basal ganglia, insula and amygdala since these structures have all been related to hypervigilance and threat monitoring.^66-70^ The amygdala is especially important for fear conditioning and threat detection.^71,72^ Lesions in the amygdala might cause individuals to misinterpret neutral sensory stimuli as threatening, leading to abnormal fear responses to sensory stimuli. The amygdala is highly interconnected with the insula,^73,74^ which monitors bodily signals such as blood pressure of heart rate (interoception) and connects these internal signals to external stimuli, shaping the emotional response to sensory stimuli.^75,76^ The frontal cortex regulates this sensory appraisal through its connections to the insula.^77^ Disruptions in frontal-insular pathways might lead to an inappropriate awareness of internal bodily signals and maladaptive affective interpretation of external stimuli, causing an exaggerated perception of threat or danger from sensory stimuli that would typically be considered non-threatening. It is important to note that the proposed attentional and emotional hypotheses are not mutually exclusive. Emotion and cognition are closely linked, and emotional states (such as anxiety) are known to affect selective attention.^78,79^ As a result, misinterpreting sensory stimuli as threatening can result in an excessive focus on—and difficulty disengaging from—otherwise neutral stimuli.

In summary, our results provide evidence that post-stroke sensory hypersensitivity is related to damage to different neural structures and white matter tracts that are involved in selective attention, sensory appraisal, and fear-related hypervigilance. As our understanding of the underlying mechanisms of post-stroke sensory hypersensitivity increases, the role of the insula, thalamus, basal ganglia, amygdala, fronto-insular tracts, and fronto-striatal tract can be understood through their involvement in other cognitive or psychosocial functions (such as multisensory integration, working memory, or coping).^80-84^

### The prevalence and evolution of post-stroke sensory hypersensitivity

In addition to providing information regarding the neural basis of post-stroke sensory hypersensitivity, this study is among the first to assess the prevalence of multi-modal post-stroke sensory hypersensitivity in a (sub)acute stroke population. Noteworthy, the prevalence of post-stroke sensory hypersensitivity in the current sample (47%) was lower than in a chronic stroke sample that also used the MESSY (75%).^6^ This might be due to methodological differences such as the self-selection bias of the chronic stroke study as well as the different in- and exclusion criteria between the two studies. Indeed, the exclusion of certain stroke types (i.e. subarachnoid haemorrhage and subdural hematoma) as well as patients with major microvascular damage or with previous strokes limits the extent to which the current sample represents the entire stroke population. On the other hand, the difference in prevalence of post-stroke sensory hypersensitivity between the acute and chronic sample might also reflect true changes in prevalence across time. For instance, it is possible that sensory hypersensitivity symptoms are not always noticeable in the subacute phase and only become apparent when participation demands increase in the chronic phase after stroke (e.g. returning to work, driving in traffic, taking part in large social gatherings). Learning more about the prevalence of post-stroke sensory hypersensitivity as well as its neural basis can greatly enhance our understanding of the underlying mechanisms of these subjective symptoms as well as help identify patients that are at risk of developing post-stroke sensory hypersensitivity. Since, there are, to our knowledge, no studies that investigate the longitudinal symptom dynamics of post-stroke sensory hypersensitivity,^3^ future research into the evolution of these symptoms is necessary. Furthermore, it remains unclear if neural mechanisms play a similar role in (sub)acute and chronic symptoms of post-stroke sensory hypersensitivity. It is possible that in the acute stages after injury, post-stroke sensory hypersensitivity is largely determined by neural mechanisms (i.e. lesion location), whereas in the chronic stages of injury psychosocial mechanisms (e.g. coping, social influences) might play a larger role. Therefore, it would be interesting for future studies to investigate the relationship between acute brain lesions and chronic sensory hypersensitivity symptoms.

### Limitation of the current study

Firstly, to increase statistical power of the multivariate lesion-symptom mapping analysis, only voxels that were lesioned in at least five participants were included in the analysis.^18,85^ The lesions of our sample overlapped in middle cerebral artery regions (in the left and right hemisphere) but did not reach sufficient coverage in other areas that might be of interest (such as the frontal or sensory cortices). As a result, the conclusions of this study are spatially limited and biased towards the regions in which we had sufficient lesion coverage.^86^ This limited lesion coverage is not specific to our study but is a common occurrence in lesion-symptom mapping studies.^48,87,88^ It does, however, limit the sensitivity of our analyses and impedes us from studying the relationship between post-stroke sensory hypersensitivity and some large-scale neural networks. In addition, it makes it hard to draw conclusions about a hemispheric dominance for post-stroke sensory hypersensitivity. When considering both hemispheres in our analyses, we only found evidence for significant results in the left hemisphere. Since post-stroke sensory hypersensitivity has been reported after both left- and right hemispheric stroke,^4,6^ a strict hemispheric dominance for post-stroke sensory hypersensitivity seems unlikely. However, this does not rule out hemispheric-specific differences in how particular neural regions relate to post-stroke sensory hypersensitivity. To explore whether the significant results in the left hemisphere could be attributed to a difference in lesion volume and lesion distribution between the included left- and right-hemispheric lesions, we flipped all left hemispheric lesions on the right-hemisphere (similar to Meyer *et al*.^48^ and Wang *et al*.^49^). Significant results in the thalamus and putamen remained, indicating that these results were not merely driven by the limited number of left-lesioned control patients. However, clusters in the insula and amygdala no longer reached significance, possibly due to underlying structural and functional asymmetries. A disadvantage of simply flipping a lesion from one hemisphere onto the other is that it does not consider possibly structural or functional asymmetries. Indeed, there is emerging evidence of structural and functional asymmetries of the insula and amygdala that might explain the lack of significant right-hemispheric results in these clusters^89,90^ For instance, the left amygdala is activated more often than the right amygdala in emotional processing.^91^ Also, right insular lesions have been linked to reduced sympathetic activation (i.e. hypoarousal), whereas left insular lesions have been linked to reduced parasympathetic regulation (i.e. hyperarousal).^92^ In addition, it is crucial to note that the lack of evidence for a statistically significant result between the right insula and amygdala and post-stroke sensory hypersensitivity does not equate to evidence for the absence of such a relationship. Our study may have been underpowered due to its modest sample size, making it more challenging to detect effects in regions where lesion-symptom relationships may be more variable or subtle. Further research on these asymmetries as well as lesion-symptom studies in a larger stroke sample is needed to understand the potential lateralization and involvement of the amygdala and insula in post-stroke sensory hypersensitivity.

A second limitation of this study is that we focused solely on the structural consequences of stroke without considering influences of neuroplasticity or recovery.^93^ Previous research has shown that within the first few days after stroke the brain engages in functional reorganization.^94-96^ This functional reorganization includes both lesion-related functional changes as well as secondary compensatory responses (where other brain regions take over the functions performed by the lesioned area). These effects could not be explored using the current methodology. Future studies could make use of modern techniques such as indirect functional connectivity mapping.^97^ This method involves overlaying lesions delineated on routine clinical imaging onto a publicly available dataset of resting state fMRI data from neurotypical adults. This technique can identify a network of brain regions that are functionally related to the brain lesion and might display abnormalities post-injury. These identified networks are then compared between patients with and without a certain symptom to investigate whether functional abnormalities in certain regions are related to behaviour.^98^ However, to date, this technique does not predict behavioural data better than standard lesion-symptom mapping or indirect structural disconnection mapping,^23^ indicating that direct functional measures are still essential. Therefore, to provide insight on the functional neural mechanisms of post-stroke sensory hypersensitivity, future studies should conduct fMRI research, possibly combined with lesion-symptom mapping.

A last limitation, that is not specific to our study but to lesion-symptom mapping in general, is that lesion-symptom mapping techniques induce a spatial misplacement of their results (oriented towards the middle and posterior arteries).^41,99^ To gain more certainty about the reliability of the spatial location of our results, we encourage replication studies using larger heterogenous samples.^86^ As an additional benefit, studying a larger sample might provide important information about whether the neuroanatomy of post-stroke sensory hypersensitivity differs according to the sensory modality that is affected, as well as if there are differences in neuroanatomy between uni-modal and multi-modal sensory hypersensitivity. Due to the limited number of patients with (uni-modal) sensory hypersensitivity, our sample did not allow for such analyses. Lastly, having a larger sample could allow researchers to conduct multivariate analyses that integrate data regarding interoceptive awareness, cognition (e.g. selective attention), psychosocial factors (e.g. anxiety, threat monitoring) as well as lesion-behavior mapping data. This approach would provide a deeper insight into the role of brain damage relative to other mechanisms and explore potential interactions between these factors.

## Conclusion

In conclusion, this study provides evidence for a relationship between post-stroke sensory hypersensitivity and damage to the insula, putamen, amygdala and thalamus as well as different white matter tracts (fronto-insular tracts and the fronto-striatal tract). This provides us with important information about which patients are at risk for developing post-stroke sensory hypersensitivity but can also teach us something about which brain regions play a role in sensory sensitivity, making it of interest for other clinical groups.

## Supplementary Material

fcaf176_Supplementary_Data

## Data Availability

The data that support the findings of this study are available upon request from the corresponding author or are publicly available at https://doi.org/10.6084/m9.figshare.28322135.

## References

[1] Greven CU, Lionetti F, Booth C, et al Sensory processing sensitivity in the context of environmental sensitivity: A critical review and development of research agenda. Neurosci Biobehav Rev. 2019;98(January):287–305. 30639671 10.1016/j.neubiorev.2019.01.009

[2] Ward J . Individual differences in sensory sensitivity: A synthesizing framework and evidence from normal variation and developmental conditions. Cogn Neurosci. 2019;10(3):139–157. 30526338 10.1080/17588928.2018.1557131

[3] Thielen H, Tuts N, Welkenhuyzen L, Huenges Wajer IMC, Lafosse C, Gillebert CR. Sensory sensitivity after acquired brain injury: A systematic review. J Neuropsychol. 2022;17(1):1–31. 35773750 10.1111/jnp.12284

[4] Thielen H, Huenges Wajer IMC, Tuts N, Welkenhuyzen L, Lafosse C, Gillebert CR. The multi-modal evaluation of sensory sensitivity (MESSY): Assessing a commonly missed symptom of acquired brain injury. Clin Neuropsychol. 2023;0(0):1–35. 10.1080/13854046.2023.2219024 37291083

[5] Chung SM, Song BK. Evaluation of sensory processing abilities following stroke using the adolescent/adult sensory profile: Implications for individualized intervention. J Phys Ther Sci. 2016;28(10):2852–2856. 27821949 10.1589/jpts.28.2852 PMC5088140

[6] Thielen H, Tuts N, Lafosse C, Gillebert CR. The neuroanatomy of poststroke subjective sensory hypersensitivity. Cogn Behav Neurol. 2023;36(2):68–84. 37026772 10.1097/WNN.0000000000000341

[7] Alwawi DA, Dean E, Heldstab A, Lawson LM, Peltzer J, Dunn W. A qualitative study of stroke survivors’ experience of sensory changes. Can J Occup Ther. 2020;87(4):298–306. 32696659 10.1177/0008417420941975

[8] Bijlenga D, Tjon-Ka-Jie JYM, Schuijers F, Kooij JJS. Atypical sensory profiles as core features of adult ADHD, irrespective of autistic symptoms. Eur Psychiatry. 2017;43:51–57. 28371743 10.1016/j.eurpsy.2017.02.481

[9] López-Solá M, Pujol J, Wager TD, et al Altered functional magnetic resonance imaging responses to nonpainful sensory stimulation in fibromyalgia patients. Arthritis Rheumatol. 2014;66(11):3200–3209. 25220783 10.1002/art.38781 PMC4410766

[10] Ochi R, Saito S, Hiromitsu K, et al Sensory hypo- and hypersensitivity in patients with brain tumors. Brain Inj. 2022;36(8):1053–1058. 35971300 10.1080/02699052.2022.2110943

[11] Tavassoli T, Hoekstra RA, Baron-Cohen S. The sensory perception quotient (SPQ): Development and validation of a new sensory questionnaire for adults with and without autism. Mol Autism. 2014;5(1):29. 24791196 10.1186/2040-2392-5-29 PMC4005907

[12] Boucher O, Turgeon C, Champoux S, et al Hyperacusis following unilateral damage to the insular cortex: A three-case report. Brain Res. 2015;1606:102–112. 25721796 10.1016/j.brainres.2015.02.030

[13] Cantone M, Lanza G, Bella R, et al Fear and disgust: Case report of two uncommon emotional disturbances evoked by visual disperceptions after a right temporal-insular stroke. BMC Neurol. 2019;19(1):193. 31409291 10.1186/s12883-019-1417-0 PMC6693272

[14] Mak YE, Simmons KB, Gitelman DR, Small DM. Taste and olfactory intensity perception changes following left insular stroke. Behav Neurosci. 2005;119(6):1693–1700. 16420174 10.1037/0735-7044.119.6.1693

[15] Pritchard TC, Macaluso DA, Eslinger PJ. Taste perception in patients with insular cortex lesions. Behav Neurosci. 1999;113(4):663–671. 10495075

[16] Caviness VS, Makris N, Montinaro E, et al Anatomy of stroke, part I: An MRI-based topographic and volumetric system of analysis. Stroke. 2002;33(11):2549–2556. 12411641 10.1161/01.str.0000036083.90045.08

[17] Baldo JV, Ivanova MV, Herron TJ, Wilson SM, Dronkers NF. Voxel-based lesion symptom mapping. In: Pustina D, Mirman D, eds. Lesion-to-symptom mapping: Principles and tools. Neuromethods. Springer US; 2022:95–118.

[18] de Haan B, Karnath HO. A hitchhiker’s guide to lesion-behaviour mapping. Neuropsychologia. 2018;115(June 2017):5–16. 29066325 10.1016/j.neuropsychologia.2017.10.021

[19] Zhang Y, Kimberg DY, Coslett HB, Schwartz MF, Wang Z. Multivariate lesion-symptom mapping using support vector regression. Hum Brain Mapp. 2014;35(12):5861–5876. 25044213 10.1002/hbm.22590 PMC4213345

[20] Gillebert CR, Mantini D. Functional connectivity in the normal and injured brain. Neurosci Rev J Bringing Neurobiol Neurol Psychiatry. 2013;19(5):509–522. 10.1177/1073858412463168 23064084

[21] Gleichgerrcht E, Wilmskoetter J, Bonilha L. Connectome-based lesion-symptom mapping using structural brain imaging. In: Pustina D, Mirman D, eds. Lesion-to-symptom mapping: Principles and tools. Neuromethods. Springer US; 2022:167–180.

[22] Kuceyeski A, Boes A. Lesion-network mapping: From a topologic to hodologic approach. In: Pustina D, Mirman D, eds. Lesion-to-symptom mapping: Principles and tools. Neuromethods. Springer US; 2022:149–166.

[23] Salvalaggio A, De Filippo De Grazia M, Zorzi M, Thiebaut de Schotten M, Corbetta M. Post-stroke deficit prediction from lesion and indirect structural and functional disconnection. Brain. 2020;143(7):2173–2188. 32572442 10.1093/brain/awaa156 PMC7363494

[24] Foulon C, Cerliani L, Kinkingnéhun S, et al Advanced lesion symptom mapping analyses and implementation as BCBtoolkit. GigaScience. 2018;7(3):1–17. 10.1093/gigascience/giy004 PMC5863218 29432527

[25] Sperber C, Griffis J, Kasties V. Indirect structural disconnection-symptom mapping. Brain Struct Funct. 2022;227(9):3129–3144. 36048282 10.1007/s00429-022-02559-x

[26] Fazekas F, Chawluk JB, Alavi A, Hurtig HI, Zimmerman RA. MR signal abnormalities at 1.5 T in Alzheimer’s dementia and normal aging. AJR Am J Roentgenol. 1987;149(2):351–356. 3496763 10.2214/ajr.149.2.351

[27] Dalemans R, Wade DT, van den Heuvel WJ, de Witte LP. Facilitating the participation of people with aphasia in research: A description of strategies. Clin Rehabil. 2009;23(10):948–959. 19570814 10.1177/0269215509337197

[28] Huygelier H, Schraepen B, Demeyere N, Gillebert CR. The Dutch version of the Oxford cognitive screen (OCS-NL): Normative data and their association with age and socio-economic status. Aging Neuropsychol Cogn. 2019;27(5):765–786. 10.1080/13825585.2019.1680598 31684814

[29] Huygelier H, Schraepen B, Miatton M, et al The Dutch Oxford cognitive screen (OCS-NL): Psychometric properties in Flemish stroke survivors. Neurol Sci. 2022;43(11):6349–6358. 35971043 10.1007/s10072-022-06314-2

[30] Demeyere N, Riddoch MJ, Slavkova ED, Bickerton WL, Humphreys GW. The Oxford cognitive screen (OCS): Validation of a stroke-specific short cognitive screening tool. Psychol Assess. 2015;27:883–894. 25730165 10.1037/pas0000082

[31] Brott T, Adams HP, Olinger CP, et al Measurements of acute cerebral infarction: A clinical examination scale. Stroke. 1989;20(7):864–870. 2749846 10.1161/01.str.20.7.864

[32] RStudio: Integrated Development for R [Computer Software]. Version 2023.09.0+463. RStudio, PBC; 2023. https://www.rstudio.com/

[33] MATLAB [Computer Software]. Version R2018b. The MathWorks Inc; 2018.

[34] Holm S . A simple sequentially rejective multiple test procedure. Scand J Stat. 1979;6(2):65–70.

[35] Moore MJ, Jenkinson M, Griffanti L, Huygelier H, Gillebert CR, Demeyere N. A comparison of lesion mapping analyses based on CT versus MR imaging in stroke. Neuropsychologia. 2023;184:108564. 37068585 10.1016/j.neuropsychologia.2023.108564 PMC10933788

[36] Biesbroek JM, Kuijf HJ, Weaver NA, Zhao L, Duering M, Biessels GJ. Brain infarct segmentation and registration on MRI or CT for lesion-symptom mapping. J Vis Exp. 2019;2019(151):1–16. 10.3791/59653 31609325

[37] Lugtmeijer S, Geerligs L, de Leeuw FE, de Haan EHF, Kessels RPC. Are visual working memory and episodic memory distinct processes? Insight from stroke patients by lesion-symptom mapping. Brain Struct Funct. 2021;226(6):1713–1726. 33914126 10.1007/s00429-021-02281-0 PMC8203519

[38] Zhao L, Biesbroek JM, Shi L, et al Strategic infarct location for post-stroke cognitive impairment: A multivariate lesion-symptom mapping study. J Cereb Blood Flow Metab. 2018;38(8):1299–1311. 28895445 10.1177/0271678X17728162 PMC6092771

[39] DeMarco AT, Turkeltaub T. A multivariate lesion symptom mapping toolbox and examination of lesion-volume biases and correction methods in lesion-symptom mapping. Hum Brain Mapp. 2018;39(11):4169–4182. 29972618 10.1002/hbm.24289 PMC6647024

[40] Karnath HO, Sperber C, Wiesen D, de Haan B. Lesion-behavior mapping in cognitive neuroscience: A practical guide to univariate and multivariate approaches. Neuromethods. 2020;151(April 2019):209–238.

[41] Sperber C, Wiesen D, Karnath H. An empirical evaluation of multivariate lesion behaviour mapping using support vector regression. Hum Brain Mapp. 2018;40(5):1381–1390. 30549154 10.1002/hbm.24476 PMC6865618

[42] Teghipco A, Newman-Norlund R, Gibson M, et al Stable multivariate lesion symptom mapping. Apert Neuro. 2024:4:10.52294/001c.117311. 10.52294/001c.117311 PMC11449259 39364269

[43] Faulkner JW, Wilshire CE. Mapping eloquent cortex: A voxel-based lesion-symptom mapping study of core speech production capacities in brain tumour patients. Brain Lang. 2020;200:104710. 31739187 10.1016/j.bandl.2019.104710

[44] Mirman D, Landrigan JF, Kokolis S, Verillo S, Ferrara C, Pustina D. Corrections for multiple comparisons in voxel-based lesion-symptom mapping. Neuropsychologia. 2018;115(March 2017):112–123. 28847712 10.1016/j.neuropsychologia.2017.08.025 PMC5826816

[45] Rolls ET, Huang CC, Lin CP, Feng J, Joliot M. Automated anatomical labelling atlas 3. NeuroImage. 2020;206:116189. 31521825 10.1016/j.neuroimage.2019.116189

[46] Rojkova K, Volle E, Urbanski M, Humbert F, Dell’Acqua F, Thiebaut de Schotten M. Atlasing the frontal lobe connections and their variability due to age and education: A spherical deconvolution tractography study. Brain Struct Funct. 2016;221(3):1751–1766. 25682261 10.1007/s00429-015-1001-3

[47] Weaver NA, Lim JS, Schilderinck J, et al Strategic infarct locations for poststroke depressive symptoms: A lesion- and disconnection-symptom mapping study. Biol Psychiatry Cogn Neurosci Neuroimaging. 2023;8(4):387–396. 34547548 10.1016/j.bpsc.2021.09.002

[48] Meyer S, Kessner SS, Cheng B, et al Voxel-based lesion-symptom mapping of stroke lesions underlying somatosensory deficits. NeuroImage Clin. 2016;10:257–266. 26900565 10.1016/j.nicl.2015.12.005 PMC4724038

[49] Wang J, Gu M, Xiao L, et al Association of lesion location and fatigue symptoms after ischemic stroke: A VLSM study. Front Aging Neurosci. 2022;14:902604. 35847675 10.3389/fnagi.2022.902604 PMC9277067

[50] Green SA, Hernandez L, Bookheimer SY, Dapretto M. Reduced modulation of thalamocortical connectivity during exposure to sensory stimuli in ASD. Autism Res. 2017;10(5):801–809. 27896947 10.1002/aur.1726 PMC5444966

[51] Green SA, Hernandez L, Tottenham N, Krasileva K, Bookheimer SY, Dapretto M. Neurobiology of sensory overresponsivity in youth with autism spectrum disorders. JAMA Psychiatry. 2015;72(8):778–786. 26061819 10.1001/jamapsychiatry.2015.0737 PMC4861140

[52] Green SA, Rudie JD, Colich NL, et al Overreactive brain responses to sensory stimuli in youth with autism spectrum disorders. J Am Acad Child Adolesc Psychiatry. 2013;52(11):1158–1172. 24157390 10.1016/j.jaac.2013.08.004 PMC3820504

[53] Shepherd D, Landon J, Kalloor M, Theadom A, Grp BR. Clinical correlates of noise sensitivity in patients with acute TBI. BRAIN Inj. 2019;33(8):1050–1058. 31007081 10.1080/02699052.2019.1606443

[54] Thielen H, Gillebert CR. Sensory sensitivity: Should we consider attention in addition to prediction? Cogn Neurosci. 2019;10(3):158–160. 30898072 10.1080/17588928.2019.1593125

[55] Panagiotidi M, Overton PG, Stafford T. The relationship between ADHD traits and sensory sensitivity in the general population. Compr Psychiatry. 2018;80:179–185. 29121555 10.1016/j.comppsych.2017.10.008

[56] Panagopoulos VN, Greene DJ, Campbell MC, Black KJ. Towards objectively quantifying sensory hypersensitivity: A pilot study of the “Ariana effect.”. PeerJ. 2013;1:e121. 23940834 10.7717/peerj.121 PMC3740136

[57] Menon V, Uddin LQ. Saliency, switching, attention and control: A network model of insula function. Brain Struct Funct. 2010;214(5–6):655–667. 20512370 10.1007/s00429-010-0262-0 PMC2899886

[58] Torrico TJ, Munakomi S. Neuroanatomy, thalamus. StatPearls Publishing; 2023. 31194341

[59] John YJ, Zikopoulos B, Bullock D, Barbas H. The emotional gatekeeper: A computational model of attentional selection and suppression through the pathway from the amygdala to the inhibitory thalamic reticular nucleus. PLoS Comput Biol. 2016;12(2):e1004722. 26828203 10.1371/journal.pcbi.1004722 PMC4734702

[60] Zikopoulos B, Barbas H. Circuits for multisensory integration and attentional modulation through the prefrontal cortex and the thalamic reticular nucleus in primates. Rev Neurosci. 2007;18(6):417–438. 18330211 10.1515/revneuro.2007.18.6.417 PMC2855189

[61] Nakajima M, Schmitt LI, Halassa MM. Prefrontal cortex regulates sensory filtering through a Basal Ganglia-to-Thalamus pathway. Neuron. 2019;103(3):445–458.e10. 31202541 10.1016/j.neuron.2019.05.026 PMC6886709

[62] Bonato M, Priftis K, Umiltà C, Zorzi M. Computer-based attention-demanding testing unveils severe neglect in apparently intact patients. Behav Neurol. 2013;26(3):179–181. 22713418 10.3233/BEN-2012-129005 PMC5214525

[63] Demeyere N, Haupt M, Webb SS, et al Introducing the tablet-based Oxford cognitive screen-plus (OCS-plus) as an assessment tool for subtle cognitive impairments. Sci Rep. 2021;11(1):8000. 33846501 10.1038/s41598-021-87287-8 PMC8041764

[64] Gillebert CR, Mantini D, Thijs V, Sunaert S, Dupont P, Vandenberghe R. Lesion evidence for the critical role of the intraparietal sulcus in spatial attention. Brain J Neurol. 2011;134(Pt 6):1694–1709. 10.1093/brain/awr085 21576110

[65] Thielen H, Welkenhuyzen L, Tuts N, et al Why am I overwhelmed by bright lights? The behavioural mechanisms of post-stroke visual hypersensitivity. Neuropsychologia. 2024;198:108879. 38570111 10.1016/j.neuropsychologia.2024.108879

[66] Costanzo ME, Jovanovic T, Pham D, et al White matter microstructure of the uncinate fasciculus is associated with subthreshold posttraumatic stress disorder symptoms and fear potentiated startle during early extinction in recently deployed service members. Neurosci Lett. 2016;618:66–71. 26923670 10.1016/j.neulet.2016.02.041

[67] Kirouac GJ. The paraventricular nucleus of the thalamus as an integrating and relay node in the brain anxiety network. Front Behav Neurosci. 2021;15:1–14. 10.3389/fnbeh.2021.627633 PMC7959748 33732118

[68] Somerville LH, Whalen PJ, Kelley WM. Human bed nucleus of the stria terminalis indexes hypervigilant threat monitoring. Biol Psychiatry. 2010;68(5):416–424. 20497902 10.1016/j.biopsych.2010.04.002 PMC2921460

[69] Stoffers D, Altena E, van der Werf YD, et al The caudate: A key node in the neuronal network imbalance of insomnia? Brain. 2014;137(2):610–620. 24285642 10.1093/brain/awt329 PMC3914473

[70] Šimić G, Tkalčić M, Vukić V, et al Understanding emotions: Origins and roles of the amygdala. Biomolecules. 2021;11(6):823. 34072960 10.3390/biom11060823 PMC8228195

[71] LeDoux J . The amygdala. Curr Biol. 2007;17(20):R868–R874. 17956742 10.1016/j.cub.2007.08.005

[72] Davis M, Whalen PJ. The amygdala: Vigilance and emotion. Mol Psychiatry. 2001;6(1):13–34. 11244481 10.1038/sj.mp.4000812

[73] Ghaziri J, Tucholka A, Girard G, et al Subcortical structural connectivity of insular subregions. Sci Rep. 2018;8(1):8596. 29872212 10.1038/s41598-018-26995-0 PMC5988839

[74] Chen J, Gao Y, Bao ST, et al Insula→amygdala and Insula→thalamus pathways are involved in comorbid chronic pain and depression-like behavior in mice. J Neurosci. 2024;44(15):e2062232024. 38453468 10.1523/JNEUROSCI.2062-23.2024 PMC11007474

[75] Caseras X, Murphy K, Mataix-Cols D, et al Anatomical and functional overlap within the insula and anterior cingulate cortex during interoception and phobic symptom provocation. Hum Brain Mapp. 2013;34(5):1220–1229. 22162203 10.1002/hbm.21503 PMC6869871

[76] Zaki J, Davis JI, Ochsner KN. Overlapping activity in anterior insula during interoception and emotional experience. NeuroImage. 2012;62(1):493–499. 22587900 10.1016/j.neuroimage.2012.05.012 PMC6558972

[77] Namkung H, Kim SH, Sawa A. The insula: An underestimated brain area in clinical neuroscience, psychiatry, and neurology. Trends Neurosci. 2017;40(4):200–207. 28314446 10.1016/j.tins.2017.02.002 PMC5538352

[78] Cisler JM, Koster EHW. Mechanisms of attentional biases towards threat in the anxiety disorders: An integrative review. Clin Psychol Rev. 2010;30(2):203–216. 10.1016/j.cpr.2009.11.003 PMC2814889 20005616

[79] Okon-Singer H, Hendler T, Pessoa L, Shackman AJ. The neurobiology of emotion–cognition interactions: Fundamental questions and strategies for future research. Front Hum Neurosci. 2015;9:58. 25774129 10.3389/fnhum.2015.00058 PMC4344113

[80] Alvarez RP, Kirlic N, Misaki M, et al Increased anterior insula activity in anxious individuals is linked to diminished perceived control. Transl Psychiatry. 2015;5(6):e591. 26125154 10.1038/tp.2015.84 PMC4490294

[81] Baier B, Karnath HO, Dieterich M, Birklein F, Heinze C, Muller NG. Keeping memory clear and stable–the contribution of human basal ganglia and prefrontal cortex to working memory. J Neurosci. 2010;30(29):9788–9792. 20660261 10.1523/JNEUROSCI.1513-10.2010 PMC6632833

[82] Brunert D, Rothermel M. Cortical multisensory integration—A special role of the agranular insular cortex? Pflüg Arch—Eur J Physiol. 2020;472(6):671–672. 10.1007/s00424-020-02400-6 PMC7293668 32458084

[83] Toller G, Mandelli ML, Cobigo Y, et al Right uncinate fasciculus supports socioemotional sensitivity in health and neurodegenerative disease. NeuroImage Clin. 2022;34:102994. 35487131 10.1016/j.nicl.2022.102994 PMC9125782

[84] Tyll S, Budinger E, Noesselt T. Thalamic influences on multisensory integration. Commun Integr Biol. 2011;4(4):378–381. 21966551 10.4161/cib.4.4.15222 PMC3181501

[85] Sperber C, Karnath HO. Statistical considerations in voxel-based lesion-behavior mapping. In: Pustina D, Mirman D, eds. Lesion-to-symptom mapping: Principles and tools. Neuromethods. Springer US; 2022:119–133.

[86] Moore MJ, Demeyere N, Rorden C, Mattingley JB. Lesion mapping in neuropsychological research: A practical and conceptual guide. Cortex. 2023;170:38–52. 37940465 10.1016/j.cortex.2023.10.001 PMC11474248

[87] Feldman SBZ, Soroker N, Levy DA. Lesion-behavior mapping indicates a strategic role for parietal substrates of associative memory. Cortex. 2023;167:148–166. 37562150 10.1016/j.cortex.2023.06.016

[88] Oostra KM, Van Bladel A, Vanhoonacker ACL, Vingerhoets G. Damage to fronto-parietal networks impairs motor imagery ability after stroke: A voxel-based lesion symptom mapping study. Front Behav Neurosci. 2016;10:10. 26869894 10.3389/fnbeh.2016.00005 PMC4740776

[89] Ocklenburg S, Peterburs J, Mundorf A. Hemispheric asymmetries in the amygdala: A comparative primer. Prog Neurobiol. 2022;214:102283. 35533810 10.1016/j.pneurobio.2022.102283

[90] Chiarello C, Vazquez D, Felton A, Leonard CM. Structural asymmetry of anterior Insula: Behavioral correlates and individual differences. Brain Lang. 2013;126(2):109–122. 23681069 10.1016/j.bandl.2013.03.005 PMC3722256

[91] Baas D, Aleman A, Kahn RS. Lateralization of amygdala activation: A systematic review of functional neuroimaging studies. Brain Res Rev. 2004;45(2):96–103. 15145620 10.1016/j.brainresrev.2004.02.004

[92] Holtmann O, Franz M, Mönig C, et al Lateralized deficits in arousal processing after insula lesions: Behavioral and autonomic evidence. Cortex J Devoted Study Nerv Syst Behav. 2022;148:168–179. 10.1016/j.cortex.2021.12.013 35180480

[93] Wilson SM . Lesion-symptom mapping in the study of spoken language understanding. Lang Cogn Neurosci. 2017;32(7):891–899. 29051908 10.1080/23273798.2016.1248984 PMC5642290

[94] Grefkes C, Fink GR. Recovery from stroke: Current concepts and future perspectives. Neurol Res Pract. 2020;2(1):17. 33324923 10.1186/s42466-020-00060-6 PMC7650109

[95] Rehme AK, Eickhoff SB, Wang LE, Fink GR, Grefkes C. Dynamic causal modeling of cortical activity from the acute to the chronic stage after stroke. NeuroImage. 2011;55(3):1147–1158. 21238594 10.1016/j.neuroimage.2011.01.014 PMC8053821

[96] Rehme AK, Fink GR, von Cramon DY, Grefkes C. The role of the contralesional motor cortex for motor recovery in the early days after stroke assessed with longitudinal FMRI. Cereb Cortex. 2011;21(4):756–768. 20801897 10.1093/cercor/bhq140

[97] Joutsa J, Darby RR, Fox MD. Lesion network mapping using resting-state functional connectivity MRI. In: Pustina D, Mirman D, eds. Lesion-to-symptom mapping: Principles and tools. Neuromethods. Springer US; 2022:181–198.

[98] Boes AD . Lesion network mapping: Where do we go from here? Brain. 2021;144(1):e5. 33212509 10.1093/brain/awaa350 PMC8453285

[99] Mah YH, Husain M, Rees G, Nachev P. Human brain lesion-deficit inference remapped. Brain J Neurol. 2014;137(Pt 9):2522–2531. 10.1093/brain/awu164 PMC4132645 24974384

