# Supplementary material for: Post-stroke sensory hypersensitivity: insights from lesion-symptom and disconnection mapping
Source: Brain Commun. 2025 May 6;7(3):fcaf176. doi: 10.1093/braincomms/fcaf176 (PMC12081950; doi:10.1093/braincomms/fcaf176)
Supplement: fcaf176_Supplementary_Data [file fcaf176_supplementary_data.docx]

## Supplementary materials

**Supplementary Table 1. Characteristics of the patients with and without post-stroke sensory hypersensitivity (n = 103).**

|  | Patients  without  post-stroke sensory hypersensitivity | Patients  with  post-stroke sensory hypersensitivity | Results of non-parametric  Wilcoxon rank-sum test | |
| --- | --- | --- | --- | --- |
|  |  |  | *W* | Adjusted *p* |
| Number of patients | 55 | 48 |  |  |
| Age: mean (sd), in years | 69 (12) | 62 (15) | 1691 | .16 |
| Age range: in years | 29 - 89 | 26 - 90 |  |  |
| Number of male patients (%) | 35 (64%) | 28 (58%) |  |  |
| Number of patients who completed higher education^^[[1]](#footnote-1)^^ (%) | 15 (28%) | 19 (40%) |  |  |
| Number of patients with an ischemic / haemorrhagic stroke (%) | 48 (87%) / 7 (13%) | 35 (73%) / 13 (27%) |  |  |
| Lesioned hemisphere:  left / right / bilateral:  number of patients (%) | 23 (42%) / 26 (47%) / 6 (11%) | 21 (44%) / 25 (52%) / 2 (4%) |  |  |
| Lesion volume: mean (sd), in cc | 41 (53) | 36 (53) | 1377.5 | 1 |
| Time between MESSY completion and clinical imaging: mean (sd), in days | 11 (12) | 26 (33) | 924 | .11 |
| Time between stroke onset and clinical imaging:  mean (sd), in days | 4 (8) | 7 (16) | 1287 | 1 |
| MESSY Total Score  Possible range [30-150] | 44 (13) | 65 (17) | 428.5 | < . 01 |
| NIHSS score: mean (sd) | 4 (3) | 5 (4) | 707.5 | 1 |

Sd: standard deviation. Higher education: at least a bachelor degree awarded by a college or university. Cc: cubic centimetre. *P* values were adjusted for multiple comparisons using a Holm correction.^1^ There were on average 9 days between MESSY completion and the completion of the NIHSS. The NIHSS scores of 17 patients with post-stroke sensory hypersensitivity and 5 patients without post-stroke sensory hypersensitivity were missing.

**Supplementary Table 2 The cognitive profile of the stroke patients with and without post-stroke sensory hypersensitivity assessed using the OCS-NL (n = 95).**

| OCS subtest per cognitive domain | Range of possible  scores | Incidence of impairment per group (%) | | Median score | | Results of the non-parametric Wilcoxon rank-sum test | | |
| --- | --- | --- | --- | --- | --- | --- | --- | --- |
|  |  | Without SH  (n = 50) | With SH  (n = 45) | Without SH  (n = 50) | With SH  (n = 45) | W | Adjusted  *p* | Effect size |
| Visual Field test | [0 - 4] | 12% | 4% | 4 | 4 | 1038 | 1 | .14 |
| **Language** |  |  |  |  |  |  |  |  |
| Picture naming | [0 - 4] | 4% | 7% | 4 | 4 | 1037.5 | 1 | .07 |
| Semantics | [0 - 3] | 6% | 7% | 3 | 3 | 1132.5 | 1 | .01 |
| Sentence reading | [0 - 15] | 12% | 4% | 15 | 15 | 937.5 | .39 | .23 |
| **Numeracy** |  |  |  |  |  |  |  |  |
| Number writing | [0 - 3] | 4% | 2% | 3 | 3 | 1229.5 | 1 | .01 |
| Calculations | [0 - 4] | 2% | 7% | 4 | 4 | 1183 | 1 | .06 |
| **Praxis** |  |  |  |  |  |  |  |  |
| Meaningless gesture imitation | [0 - 12] | 12% | 7% | 11 | 11 | 860.5 | .53 | .21 |
| **Memory** |  |  |  |  |  |  |  |  |
| Orientation | [0 - 4] | 12% | 13% | 4 | 4 | 1137 | 1 | .02 |
| Verbal memory: free recall and recognition | [0 - 4] | 16% | 18% | 3 | 4 | 972 | 1 | .13 |
| Episodic memory: recognition | [0 - 4] | 4% | 4% | 4 | 4 | 1016 | 1 | .12 |
| **Attention** |  |  |  |  |  |  |  |  |
| Broken hearts cancellation: |  |  |  |  |  |  |  |  |
| - Total score | [0-50] | 32% | 20% | 44 | 47 | 884 | .86 | .19 |
| - Object asymmetry | [-50 – 50] | 28% | 18% | 0 | 0 | 1061 | 1 | .06 |
| - Spatial asymmetry | [-20 – 20] | 26% | 16% | 0 | 0 | 1148 | 1 | .02 |
| Trail making task | [-12 – 12] | 20% | 11% | 0 | -1 | 1309.5 | 1 | .15 |

SH = Post-stroke sensory hypersensitivity. Eight stroke patients did not complete all the tasks of the OCS-NL because of motor deficits (for 3 patients), trouble understanding the instructions of the Broken hearts cancellation task (for 1 patients), and hospital dismissal (for 3 patients). These patients were removed from the analyses. *P* values were adjusted for multiple comparisons using a Holm correction. ^1^

**Supplementary analysis 1**

Left-hemispheric lesions and the left-hemispheric part of bilateral lesions were flipped upon the right hemisphere using MRIcron (see Supplementary Figure 1). Identical to the analysis on the unflipped lesions, only voxels that were lesioned in at least five participants were considered in the analysis. To control for multiple comparisons, we used a permutation-based continuous family wise error correction (with 2000 permutations, *p* = .05, and v = 10). We compared with SVR-LSM the groups of patients with and without post-stroke sensory hypersensitivity, while controlling for lesion volume by a direct total lesion volume control procedure^2^. Supplementary Figure 1 shows an overlay of the lesions (for the entire sample and the patients with and without post-stroke sensory hypersensitivity separately) as well as of the voxels that were lesioned in at least 5 participants.

The logistic regressions on the flipped lesions revealed significant clusters in the thalamus and putamen (see Supplementary Table 3 and Supplementary Figure 2).

**Supplementary Table 3. Descriptive statistics of the significant clusters on the flipped lesions identified by SVR-LSM**

|  | Number of voxels | MNI center of mass coordinates | | | Peak Z value in MNI coordinates | Anatomical location |
| --- | --- | --- | --- | --- | --- | --- |
|  |  | X | Y | Z | Z |  |
| Cluster 1 | 213 | 25 | -14 | -2 | -10 | Putamen and thalamus |

Anatomical location was determined using the Automated Anatomical Labelling Atlas 3.^3^


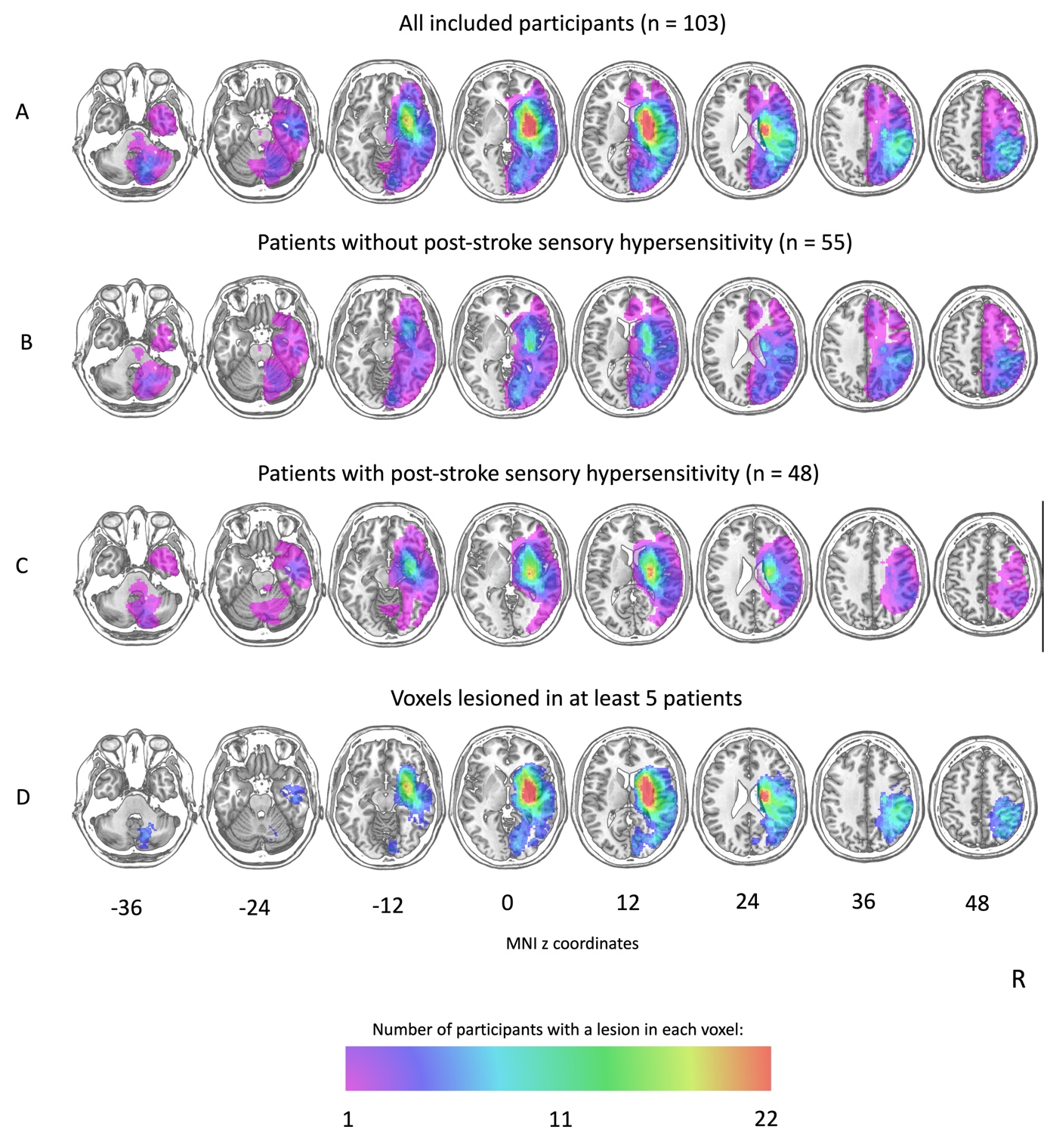


**Supplementary Figure 1. Lesion overlap maps of patient with and without post-stroke sensory hypersensitivity where all lesions are flipped upon the right hemisphere. (A)** Lesion overlap map (descriptive) of all included participants (n = 103), created by superimposing individual lesion maps onto axial slices of the T1-weighted template from the Montreal Neurological Institute (ch2-template). **(B)** Lesion overlap map (descriptive) of patients without post-stroke sensory hypersensitivity (n = 55). **(C)** Lesion overlap map (descriptive) of patients with post-stroke sensory hypersensitivity (n = 48). **(D)** Lesion overlap map displaying the voxels that were lesioned in at least five patients, i.e. the voxels that were included in the statistical analysis. The lesion maps are visualised on axial slices of the T1-weighted template from the Montreal Neurological Institute (ch2-template). The numbers refer to the MNI coordinates of the z-axis. The colour scale indicates the number of patients with a lesion in a specific voxel. The right hemisphere is presented on the right side.

**
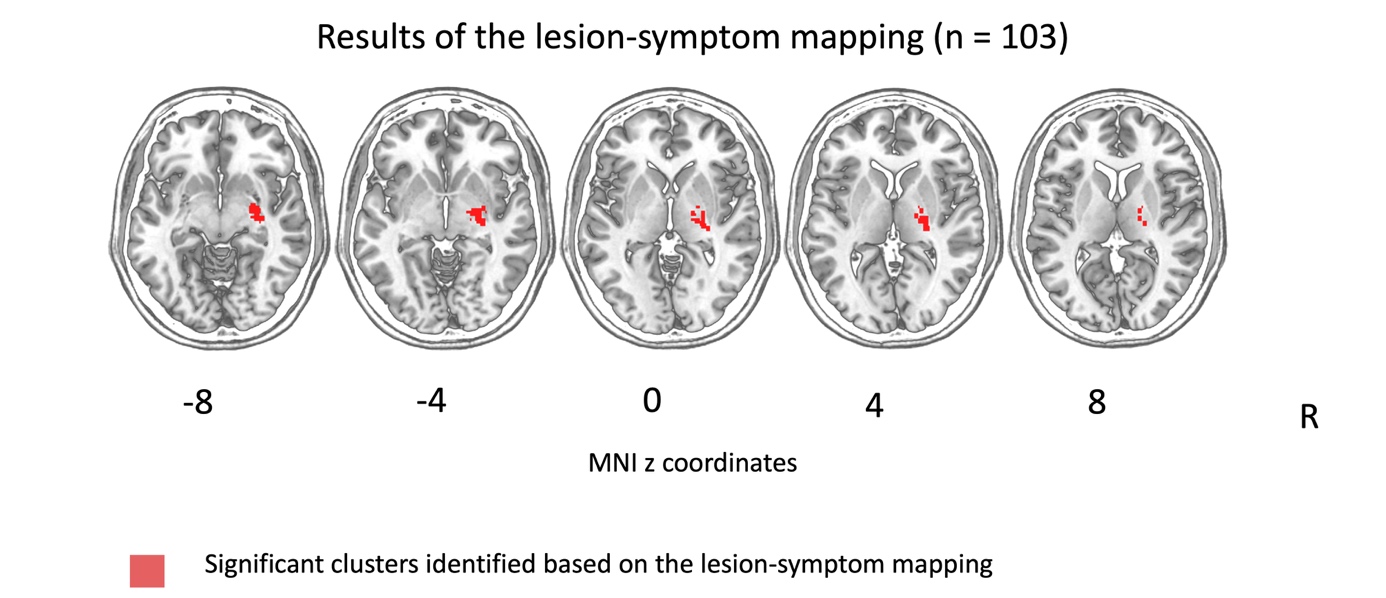
**

**Supplementary Figure 2. Statistically significant voxels identified by SVR-LSM on the flipped lesions**, showing significant clusters in the thalamus and putamen. A continuous family wise error correction for multiple comparisons was applied and the results were corrected for the effects of age and lesion volume. The lesion maps are visualised on axial slices of the T1-weighted template from the Montreal Neurological Institute (ch2-template). The numbers refer to the MNI z-coordinates of the corresponding slices. The right hemisphere is presented on the right side.

**Supplementary references**

1. The MathWorks Inc. MATLAB version: 9.5.0.1178774 (R2018b). Published online 2018. <https://www.mathworks.com>
2. Zhang Y, Kimberg DY, Coslett HB, Schwartz MF, Wang Z. Multivariate lesion-symptom mapping using support vector regression. Hum Brain Mapp. 2014;35(12):5861-5876. doi:10.1002/hbm.22590
3. Rolls ET, Huang CC, Lin CP, Feng J, Joliot M. Automated anatomical labelling atlas 3. *NeuroImage*. 2020;206:116189. doi:10.1016/j.neuroimage.2019.116189

1. The education level of one patient without post-stroke sensory hypersensitivity and one patient with post-stroke sensory hypersensitivity was not reported. [↑](#footnote-ref-1)
